# Osteonecrosis of the jaws associated with denosumab: Study of clinical and radiographic characteristics in a series of clinical cases

**DOI:** 10.4317/jced.57019

**Published:** 2020-07-01

**Authors:** Victoria I. Tofé, Leticia Bagán, José V. Bagán

**Affiliations:** 1 Oral Medicine, University of Valencia, Spain; 2 Oral Medicine of the University of Valencia, Department of Stomatology and Maxillofacial Surgery, University of Valencia, General University Hospital, Valencia, Spain

## Abstract

**Background:**

The objective of this study was to describe the clinical and radiographic characteristics of our series of medication-related osteonecrosis of the jaws (ONJ) associated with denosumab.

**Material and Methods:**

We presented 15 cases of ONJ associated with denosumab; 11 received treatment for their osteoporosis and four for cancer treatments. We recorded the most frequent clinical findings, symptoms and radiographic characteristics in our patient group, as well as local and systemic contributing factors.

**Results:**

The mean time of treatment with denosumab was 23.83 ± 12.84 months. 40% of the patients had a previous history of treatment with bisphosphonates. The most common local factor was tooth extraction (11 cases; 73.3%), and in most cases there was necrotic bone exposure (13/15, 86.67%). Osteolysis, bone sclerosis and cortical erosion were the most common radiographic findings. Stage 1 was the most frequent, present in 60% of the cases.

**Conclusions:**

In our patient group, most were in the early stages of ONJ.

** Key words:**Denosumab, osteonecrosis, jaws, radiology.

## Introduction

The first antecedent reported in the literature of maxillary osteonecrosis (ONJ) dates back to the 19th century and describes a pathology called “phosphorous necrosis” or “phossy jaw”, associated with phosphorus poisoning, with signs and symptoms similar to the ONJ (1).

In 2007, the American Association of Oral and Maxillofacial Surgeons (AAOMS) (2) defined osteonecrosis as an exposure of necrotic bone in the maxillofacial region that persists for more than 8 weeks in patients who are receiving or have received treatment with bisphosphonates and who have no previous history of radiotherapy in the jaws. However, in 2014, due to the increasing number of cases of osteonecrosis associated with other antiresorptive therapies -denosumab- and antiangiogenic therapies, the term “Osteonecrosis of the jaws related to medications” was proposed (3).

Both bisphosphonates and denosumab are predominantly indicated for the reduction of the risk of skeletal complications in patients with bone loss resulting from long-term cancer treatment, osteoporosis, or malignant bone disease (4).

Although both drugs have similar therapeutic indications, their mechanism of action is significantly different (5). While bisphosphonates must be internalized in osteoclasts to exert their effect on cells, denosumab acts in the extracellular environment (6).

In May and June 2010, the European Commission (EC) (European Medicines Agency, 2015) and the Food and Drug Administration of the United States (FDA) (Food and Drug Administration, 2010) approved the marketing and use of Prolia® for the treatment of osteoporosis in women and men with high risk of fractures, as well as for the treatment of osteopenia associated with hormonal suppression in male patients with prostate cancer. Its dose is 60 mg subcutaneously every 6 months (7).

In contrast, Xgeva® was authorized for commercialization in July 2011, being indicated for the prevention of osteoarticular pathological events (pathological fracture, bone radiotherapy, spinal cord compression or bone surgery) as well as in adults with bone metastases from solid tumors or giant cell bone tumors. Its usual dose is 120 mg subcutaneously every 4 weeks (8).

Already in 2010, Taylor *et al.* (9) published the first report that described a case of ONJ associated with denosumab and, since then, more and more clinical studies have been published that describe this complication.

In cancer patients exposed to denosumab, the risk of ONJ varies from 0.7 to 1.9% (70 to 90 cases per 10,000 patients) (3) and they have been reported to have a risk of developing ONJ similar to that of acid zoledronic (10).

In patients with osteoporosis treated with denosumab, the incidence of ONJ is lower, resulting in an even lower frequency of 0.04% (four cases per 10,000 patients) (3).

However, the number of cancer and non-cancer patients treated with ONJ-related medications and, therefore, the number of potentially adverse events seems to be constantly increasing (11).

Given this, our work aims to describe fifteen cases of ONJ in patients who have been treated with denosumab, analyze their clinical and radiographic characteristics, as well as the systemic and local factors that favor the development of these lesions.

## Material and Methods

We present a series of fifteen patients with ONJ who received denosumab for osteoporosis or cancer causes. Of these, most had osteoporosis (11 cases, 73.3%) which was treated with Prolia ®. The remaining 26.7% (4/15) were being treated with Xgeva ® due to cancer causes.

To do this, we follow the ethical guidelines of the Declaration of Helsinki. The study was approved by the Ethics Committee for Human Research of the Commission for Ethics in Experimental Research of the University of Valencia (Ref. H1441967790259).

The search system for the collection of cases was through the database of the Stomatology and Maxillofacial Surgery Service of the General University Hospital of Valencia, where those patients who had been treated with drugs whose active substance was denosumab - Prolia or Xgeva- were selected and who were diagnosed with ONJ from a clinical and radiographic point of view.

For the diagnosis of ONJ patients, the criteria described by Ruggiero *et al.* (3) were used.

Inclusion criteria were: patients who presented at least one area of mandibular / maxillary osteonecrosis and who were previously or currently treated with denosumab.

The exclusion criteria were: patients with bone lesions due to a maxillary metastasis or who had been irradiated in the cervical-facial area, those who had previously received denosumab but did not develop ONJ, and patients who developed ONJ by other antiresorptive and / or antiangiogenic agents.

For each patient we recorded the age, sex, type of disease for which denosumab was indicated, the drug administered (Prolia or Xgeva), the duration of treatment, the presence of concomitant treatment with other drugs, the presence of comorbidities and of prior treatment with bisphosphonates and local predisposing factors of ONJ.

**Figure 1:**
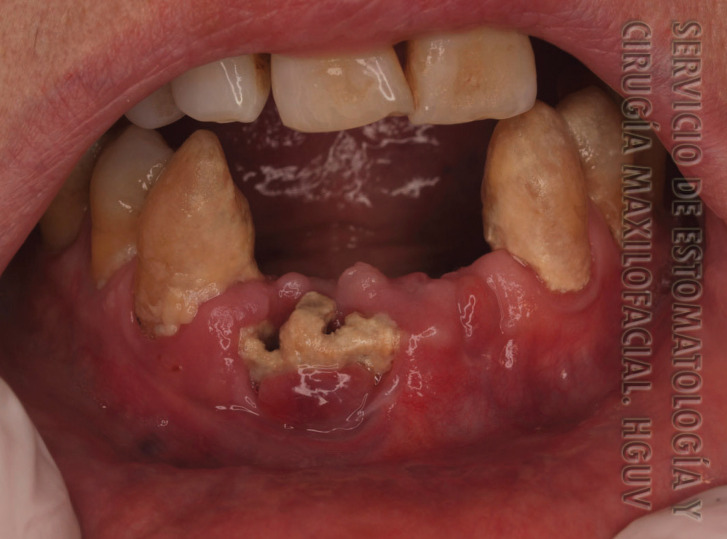
Osteonecrosis due to denosumab in the jaw in case 2.

Regarding the clinical aspect, we considered the location of the ONJ (upper jaw, jaw or both), the number of exposures, the maximum size of the exposures, the presence of pain, infection, suppuration, intraoral fistula, extraoral fistula and bone exposure (Fig. 1). Finally, we classified the 15 cases in the stages proposed by Ruggiero *et al.* (3).

**Figure 2:**
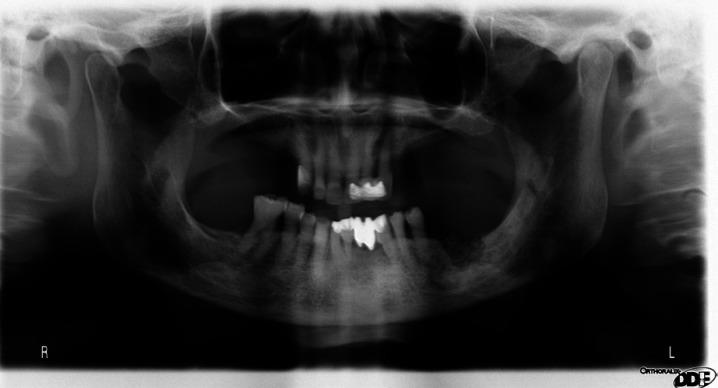
Orthopantomography of osteonecrosis in case 5.

**Figure 3:**
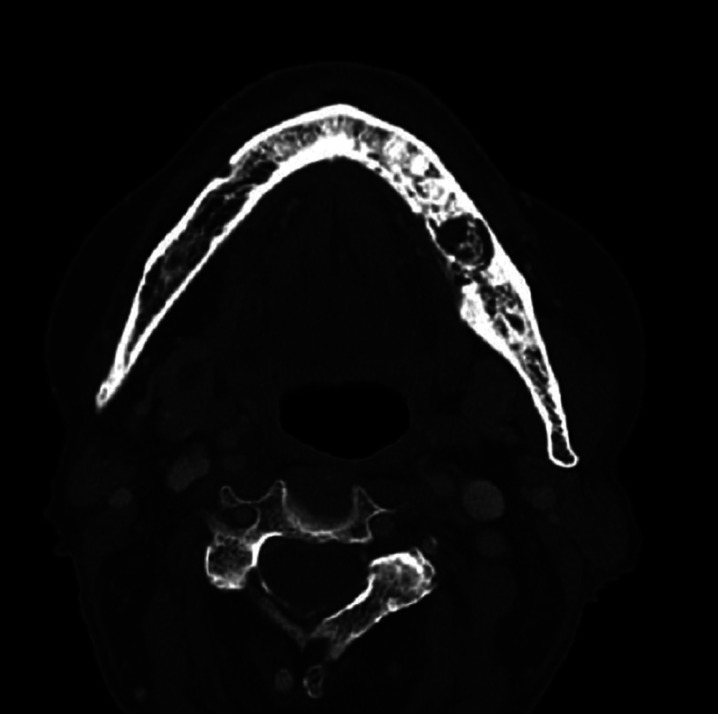
Computed tomography with evident mandibular osteolysis in case 6.

For the radiographic study, orthopantomographs and computed tomography (CT) of the patients were considered (Figs. 2,3). The presence of orosinusal involvement, osteolysis, bone sclerosis, erosion of the cortex, decrease of the mandibular canal, thickening of the mandibular cortex, mandibular fracture and bone sequestration were recorded.

As the series consisted of only 15 cases, the statistical analysis involved only the descriptive aspects of the sample, calculating the mean and standard deviation for the quantitative variables and the frequency and percentage for the categorical variables. For this we used the statistical software SPSS v. 25 for Microsoft (IBM Corp., New York, NY; formerly SPSS Inc., Chicago, IL).

## Results

The average age of our fifteen patients was 74.27 ± 9.47 years; with an age range between 57 and 89 years. Women predominated in the sample (80%).

The mean treatment time with denosumab was 30.75 ± 9.32 months for Prolia and 10 ± 4.4 months for Xgeva. 40% of the patients (6/15) had received prior therapy with oral and intravenous bisphosphonates (Table 1).

**Table 1:**
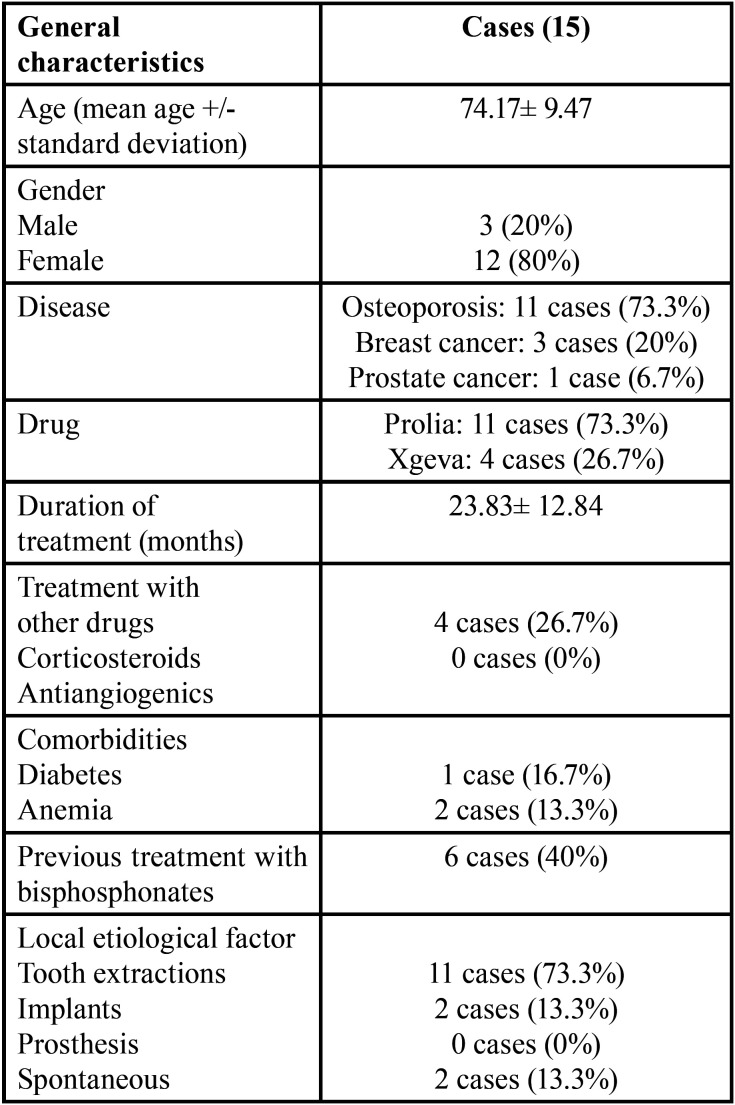
General characteristics in our series of 15 patients.

In nine of the 15 cases (60%), the ONJ was located in the jaw, and the most common local factor was tooth extraction (11 cases, 73.3%); We found only two cases associated with the placement of dental implants and two due to spontaneous causes. In six cases (40%) there was pain, two (13.3%) had an intraoral fistula, and necrotic bone exposure was presented in 13 cases (86.7%).

Stage 1 of ONJ was the most frequent since it occurred in nine cases (60%) (Table 2).

**Table 2:**
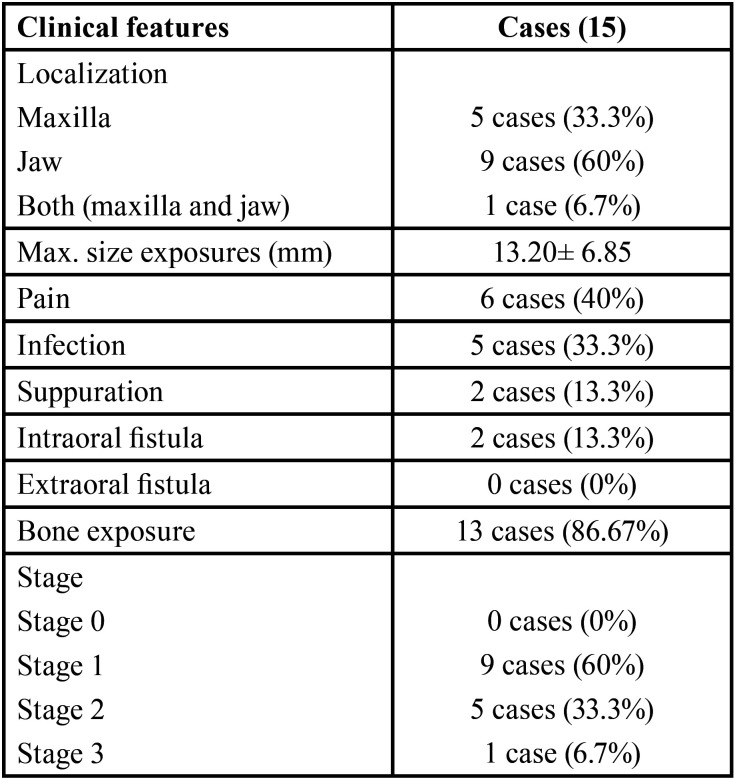
Clinical characteristics in our series of 15 patients.

Regarding the radiographic study, we found that the majority of the patients had bone lysis (12 cases, 80%), bone sclerosis (10 cases, 66.7%) and cortical erosion (10 cases, 90.9%). There was presence of bone sequestration in four cases (26.7%) (Table 3).

**Table 3:**
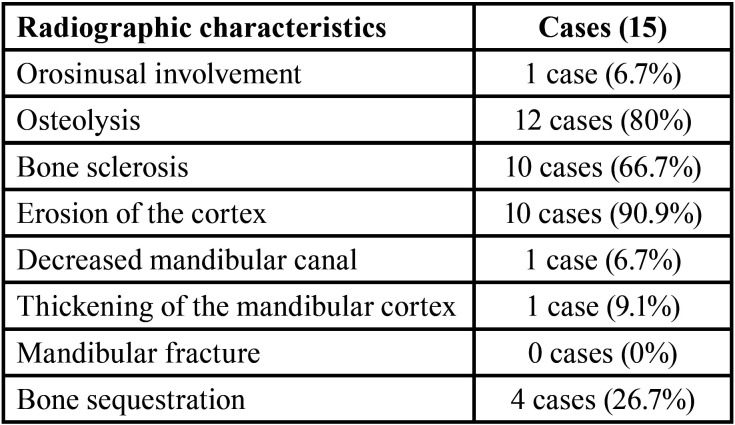
Radiographic characteristics in our series of 15 patients.

## Discussion

Denosumab is a monoclonal antibody that acts by inhibiting osteoclast activity, reducing bone resorption and increasing bone density (4).

Specifically, denosumab prevents RANKL from binding to its receptor, RANK, in the osteoclast cell membrane and osteoblastic precursors, thereby inhibiting the development, activation and survival of osteoclasts (5).

It is important to note that most of the cells that produce RANKL also produce a RANK receptor, osteoprotegerin (OPG), which acts as an antagonist of RANK signaling and osteoclastogenesis by eliminating RANKL in the extracellular environment. The relationship between RANKL and OPG determines the level of RANK activation and, therefore, the degree to which osteoclastogenesis is activated (5).

Consequently, the differentiation and function of osteoclasts are regulated by the balance between RANKL and OPG produced by osteoblasts and mesenchymal cells (13).

By joining RANKL in a similar way to OPG, denosumab prevents the interaction of RANKL and RANK, which translates into an inhibition of bone resorption (14).

Our series of osteonecrosis of the jaws associated with denosumab developed with greater prevalence in female patients (80%), with osteoporosis, who were under treatment with Prolia (73.3%). This coincides with other authors who report a greater predisposition in the female population, because the underlying diseases for which the agents are most frequently prescribed are osteoporosis and breast cancer (3).

As in other publications, the average age of the patients with ONJ in our study was 74.27 years ± 9.47 (15).

According to some authors, the risk of ONJ seems to be independent of the number of doses of denosumab or the duration of treatment (15). However, this has yet to be ratified by more research. The mean treatment time with denosumab in our case series was 23.8 ± 12.8 months.

It has been described that ONJ is a multifactorial disease since systemic and local factors are involved in its development. Within the systemic factors, it has been reported that certain comorbidities and the use of concomitant medications may increase the risk of ONJ, such as diabetes, anemia, chronic corticosteroid therapy, antiangiogenic and bisphosphonates therapy, among others (16). Of the 15 patients in our study, only one (6.7%) had diabetes and two (13.3%) had anemia.

Regarding the simultaneous use of other drugs and denosumab, Saad *et al.* in 2012 (17) described that an association can be found between the development of ONJ and the use of corticosteroids because they delay or hinder wound healing and favor the progression of lesions. In our study, four (26.7%) out of the 15 patients took corticosteroids and denosumab simultaneously.

Also, the use of other drugs such as antiangiogenic agents, when administered together with antiresorptives, is related to the appearance of osteonecrosis lesions since they can suppress vascular regeneration and, subsequently, could promote ONJ (18). However, in our study no patient was registered to have been treated with antiangiogenic agents.

We have assessed the possible contribution of previous bisphosphonate treatment, since it has been described that many patients who are currently under treatment with denosumab had previously been treated with bisphosphonates. This makes it difficult to establish which of the two drugs is more responsible for maxillary osteonecrosis, or if this could be the sum of both medications (19).

In 2018, Aljohani *et al.* (20) published a series of cases of ONJ by denosumab, where they reported that the previous use of bisphosphonates does not seem to affect the severity of ONJ by denosumab. In our study, 60% of the cases did not present prior treatment with bisphosphonates. However, it would be useful to conduct a larger sample study to conclude if both drugs together have a synergistic effect.

Dental extractions are considered to be the main trigger for developing ONJ (3). This coincides with our results, where we found that 73.3% of the cases had previously had at least one exodontics. We also observed that two patients developed osteonecrosis lesions after implant placement and that the appearance was spontaneous in two cases.

Regarding the clinical characteristics of the ONJ associated with denosumab, we have observed that the osteonecrosis lesion was mainly located in the jaw (60%), which coincides with other studies (3).

Bone exposure has been described as a key element for the diagnosis of ONJ (3). This coincides with our case series, where it was observed that bone exposure was the clinical manifestation that predominated in our sample (86.67%). Of our 15 patients, only 13.3% (2/15) had an intraoral fistula with suppuration and none had extraoral fistulas.

The most frequent stage was 1 (60%), so no signs of pain (40%) or infection (33.3%) were observed in most cases. This may be due to the fact that a large number of patients seek treatment after the onset of signs, such as when an exposed alveolar area persists after an extraction (21).

Although the diagnosis of ONJ is essentially based on the medical history and its clinical manifestations, the radiological findings are of great importance for the overall evaluation of the ONJ, determining the extent of the disease and being able to objectively define the area of necrosis, as well as for providing useful image data for subsequent surgical procedures. They are also very useful for monitoring the disease and for predicting its prognosis (22).

The imaging study of the ONJ shows manifestations such as lytic and / or sclera lesions, periosteal reactions, perforations or cortical thickening, mandibular fractures, presence of exposed necrotic bone and narrower neurovascular channels such as the nasopalatin and mandibular canal (23).

It has been described that CT and conical beam CT are useful for patients with clinical suspicion of ONJ because they give us information of initial changes in the alveolar and cortical bones of the jaw, allowing us to evaluate the presence of exposed necrotic bone, fistula formation, periostic responses and affected teeth (24).

When performing the radiographic study on orthopantomographs and CT scans of our patients, we observed that the majority presented bone lysis (80%), bone sclerosis (66.7%) and cortical erosion (90.9%) as the most frequent manifestations; this coincides with other case series of ONJ by denosumab.

Our work has some limitations since it is a retrospective study based on the analysis of a small sample size due to the low prevalence of the ONJ associated with denosumab, and it should therefore be taken as a preliminary consideration to be verified with larger series of cases and multicentric studies.

Prospective, well-controlled and larger sample studies are required to obtain more consistent criteria, both for the clinical and radiographic aspects of the ONJ by denosumab and for the local and systemic risk factors that favor its development.
